# Water sampling using modified Moore swab (MMS): the effects of sampling replicates and different media on the frequency and diversity of *Salmonella* serovars

**DOI:** 10.1128/aem.00647-25

**Published:** 2025-07-24

**Authors:** Laiorayne A. Lima, Alan D. L. Rocha, Maria L. R. Gomes, Walter E. Pereira, Patrícia E. N. Givisiez, Eric W. Brown, Marc W. Allard, Zhao Chen, Rebecca L. Bell, Magaly Toro, Jianghong Meng, Celso J. B. Oliveira

**Affiliations:** 1 Laboratório de Avaliação de Produtos de Origem Animal (LAPOA), Departamento de Zootecnia, Centro de Ciências Agrárias, Universidade Federal da Paraíba 650700 , Areia, Brazil; 2 Departamento de Ciências Fundamentais e Sociais, Centro de Ciências Agrárias, Universidade Federal da Paraíba 650688 , Areia, Brazil; 3 US Food and Drug Administration, Human Foods Program, Office of Laboratory Operations and Applied Science 116043, College Park, Maryland, USA; 4 Joint Institute for Food Safety and Applied Nutrition, and Center for Food Safety and Security Systems, University of Maryland 1068 , College Park, Maryland, USA; 5 Department of Nutrition and Food Science, University of Maryland 736324 , College Park, Maryland, USA; The Pennsylvania State University, University Park, Pennsylvania, USA

**Keywords:** agri-food systems, environmental water, food safety, irrigation water, triplicate sampling

## Abstract

*Salmonella enterica* is a significant foodborne pathogen frequently associated with produce contamination through irrigation with untreated surface water. This study investigated the effects of sampling replication and selective media on *S. enterica* serovar recovery and diversity in surface waters across Paraíba State, Brazil. Water samples (*n* = 200) were collected from 10 reservoirs using modified Moore swabs (MMSs), with three replicates per sampling site, yielding 600 samples. The prevalence of *S. enterica* was 53.5% in individual samples, increasing to 68.5% when combining results from triplicate samples. Statistical analyses demonstrated that triplicate sampling significantly enhanced both recovery rates and serovar diversity compared with single samples (*P* < 0.05). Alpha diversity metrics revealed a 39% increase in serovar richness when using triplicate versus single samples. The parallel use of multiple enrichment broths and selective agars proved essential for maximum serovar detection, as certain serovars were recovered exclusively under specific culture conditions. While triplicate sampling provided optimal detection sensitivity, duplicate sampling emerged as a cost-effective alternative, maintaining sufficient statistical power. These findings demonstrate the effectiveness of MMS combined with replicate sampling for comprehensive *S. enterica* surveillance in surface waters, with implications for water quality monitoring programs and public health risk assessment.

**IMPORTANCE:**

*Salmonella* contamination in irrigation water poses a major threat to food safety, as contaminated produce can cause widespread foodborne illness outbreaks affecting thousands of people. Current water monitoring methods often miss these hazardous bacteria, creating blind spots in our food safety systems. This research addresses a critical gap by demonstrating that taking multiple water samples from the same location, rather than just single samples, improves our ability to detect *Salmonella* contamination. The study shows that collecting three samples instead of one increases the detection rate from 54% to 69% and reveals nearly 40% more serovars. This enhanced detection capability is crucial for protecting public health, as it provides more accurate information about the occurrence of *Salmonella* in natural surface waters. Lastly, our findings provide practical guidance for improving surveillance programs worldwide, offering a cost-effective approach that significantly strengthens our defense against *Salmonella* contamination in the food supply chain.

## INTRODUCTION

*Salmonella enterica* represents a major foodborne bacterial pathogen posing significant public health risks worldwide (1). While this pathogen primarily inhabits the intestinal tract of warm-blooded animals (2), leading to the traditional association of animal-derived foods as its main source in foodborne illnesses, recent epidemiological data reveal an increasing number of salmonellosis outbreaks linked to produce consumption (3, 4). According to the 2022 Interagency Food Safety Analytics Collaboration (IFSAC) annual report, 75% of *Salmonella* illnesses in the U.S.A. originated from seven food categories. Among these, three are vegetable categories: fruits, seeded vegetables (such as tomatoes), and other produce (such as nuts) (5). This trend highlights irrigation water as a critical contamination source for *S. enterica* (3, 6), particularly untreated surface waters from rivers, lakes, ponds, and reservoirs (7, 8).

The survival and persistence of *S. enterica* in aquatic environments are influenced by multiple physicochemical parameters (9, 10). In natural water bodies, the pathogen typically occurs at low concentrations (11), significantly hampering its isolation, especially when using limited sample volumes. While sample volume directly determines the sensitivity of *Salmonella* recovery methods (11, 12), the sampling and processing of large water volumes present serious logistical challenges. Ultrafiltration techniques offer high sensitivity by concentrating microorganisms from volumes exceeding 100 L. However, they incur substantial operational costs, and their efficiency is compromised by environmental water characteristics such as high turbidity (13). As an alternative, the modified Moore swab (MMS) has emerged as a cost-effective approach for sampling larger water volumes (typically 10 L) through a simplified filtering apparatus (MMS cassette) constructed from readily available materials (14).

Despite the widespread use of MMS for *S. enterica* detection in water, critical knowledge gaps persist regarding the effect of sampling replications on recovery efficacy and the impact of selective media choices. This study aimed to evaluate how MMS sampling replications (single, duplicate, and triplicate) and varied selective media influence both the recovery frequency and serovar diversity of *S. enterica* in environmental water ecosystems.

## MATERIALS AND METHODS

### Study design

An observational study was conducted to estimate *S. enterica* prevalence and serovar diversity in reservoirs within the three largest river basins of Paraíba State, Brazil (Mamanguape, Paraíba, and Piranhas rivers). The minimum sample size (*n* = 180) was calculated as previously described (15) using an estimated *S. enterica* frequency of 20% (16), a 95% confidence level, and a 5% error margin. A total of 200 samples were collected in triplicate (R1, R2, and R3) from 10 dams and their associated rivers, resulting in 600 observations. Detailed information about sampling sites is shown in Table S1.

### Sampling and microbiological procedures

Water samplings were performed using MMSs, prepared as previously described (14). Shortly, a 0.9 m^2^ folded cheesecloth grade #90 was tightly rolled into an assembled apparatus (MMS cassette) consisting of a 10 cm-long polyvinyl chloride tube with a male-to-male coupler at one end and a connector at the other end. The assembly resulted in a filtration cassette unit (FCU), providing a cylindrical-shaped swab as a filtering matrix. FCUs were individually packed and sterilized by autoclaving. At each sampling point, three FCUs were used sequentially. Using a sterile latex tube, they were unpacked and attached to a portable peristaltic pump (CPD-201-3; MS Tecnopon Equipamentos Especiais LTDA, SP, Brazil). In each sample replicate, a volume of 10 L of water was filtered for a period of 20 min at a rate of 500 mL/min. Afterward, the swabs from each FCU were transferred aseptically into sterile containers with 250 mL of modified buffered peptone water (1.250 g of sodium chloride, 0.875 g of disodium hydrogen phosphate, and 0.375 g of potassium dihydrogen phosphate) and kept on ice during transport to the laboratory.

Microbiological isolation was performed according to the Food and Drug Administration Bacteriological Analytical Manual method (17) with minor modifications. Shortly, samples were incubated for 18–20 h at 37°C ± 0.5°C. From each sample, 100 µL and 1 mL aliquots were transferred into 9.9 mL Rappaport Vassiliadis (RV) (Oxoid, UK) and into 9 mL tetrathionate (TT) (Oxoid). After incubation at 42.5°C ± 0.5°C for 18–24 h, a loopful from each broth was plated on xylose lactose tergitol-4 agar plates (Oxoid). In parallel, 1 mL aliquots of enriched RV and TT broths were submitted to DNA extraction (18) for broth cultivation PCR (BC-PCR) assay. PCR reactions were performed in a 25 µL final master mix volume containing forward (5′ GTG AAA TTA TCG CCA CGT TCG GGC AA 3′) and reverse (5′ TCA TCG CAC CGT CAA AGG AAC C 3′) primers targeting *S. enterica invA* gene. Amplicons were electrophorized in agarose gel using a horizontal chamber (Mini SubCell GT; Bio-Rad, California, USA) at 80 V and 400 mA.

Broth samples positive for *S. enterica* by PCR were further plated onto Hektoen enteric agar (Oxoid) and bismuth sulfite (BS) agar (Oxoid). Plates were incubated at 37.0°C ± 0.5°C for 24 h. From each plate, up to three presumptive *Salmonella* colonies were further screened by biochemical tests using lysine iron agar and triple sugar iron incubated at 37.0°C ± 0.5°C overnight. Isolates showing typical *S. enterica* morphologic characteristics were transferred to tryptic soy agar and confirmed by PCR using the same primers and thermal cycling conditions applied to enrichment broths. Swabs with at least one positive isolate confirmed by PCR were considered positive.

Up to four isolates from each positive replicate were subjected to whole-genome sequencing in the scope of the GenomeTrakr initiative (https://www.fda.gov/food/whole-genome-sequencing-wgs-program/genometrakr-network). Sequencing was performed on the NextSeq 2000 platform (Illumina Inc.) with 2 × 150 bp paired-end chemistry. Whole-genome sequencing, assembly, quality control, and serotyping were performed as previously described (19). Identification of samples, accession number of their genome assemblies in National Center for Biotechnology Information, and their respective serovars can be seen in Table S2.

### Data analysis

Samples were classified as positive when at least one isolate was recovered from any of the three replicates (R1 or R2 or R3). Conversely, samples were classified as negative when no isolates were recovered in all three replicates, regardless of BC-PCR results. The sensitivity of the BC-PCR was calculated by dividing the number of positive samples detected by BC-PCR by the total number of positive samples identified by culture (considered the gold standard).

The Friedman test was used to investigate putative differences in the frequency and diversity of *Salmonella* serovars across the replicates (R1, R2, and R3), followed by paired Wilcoxon tests with Bonferroni correction for pairwise comparisons. McNemar’s test was performed to compare *Salmonella* prevalence in each replicate with the prevalence in triplicate sampling (true positive samples). Fleiss’ kappa coefficient (20) was used to evaluate the agreement across the individual sampling replicates. Cohen’s kappa test was used to measure the level of agreement between the two broths (RV and TT) used in BC-PCR. The agreement levels based on the kappa coefficient were interpreted as none (0–0.20), minimal (0.21–0.39), weak (0.40–0.59), moderate (0.60–0.79), strong (0.80–0.90), or almost perfect (above 0.90), as previously suggested (21).

**Fig 1:**
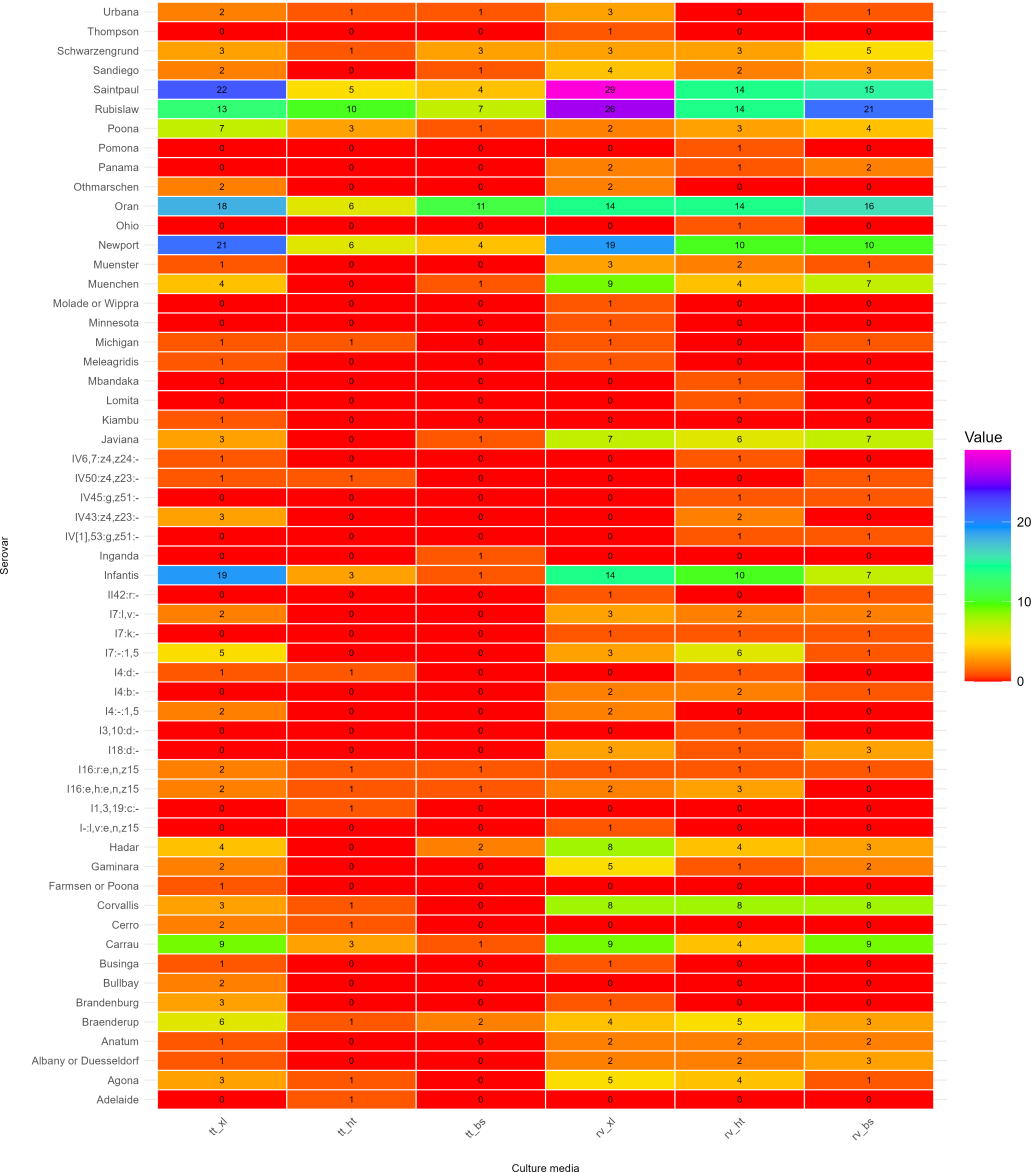
Heatmap showing the number of *Salmonella enterica* serovars recovered from surface water samples according to different enrichment broth (Rappaport-Vassiliadis [rv] and tetrathionate [tt]) and selective agar (xylose lactose tergitol-4 [xl], Hektoen [ht], and bismuth sulfite [bs]) combinations: rv_xl, tt_xl, rv_ht, tt_ht, rv_bs, and tt_bs.

The effects of replicate sampling and different enrichment media on the diversity of *Salmonella* serovars were determined using the Shannon coefficient index for alpha diversity. The abundance of *Salmonella* serovars recovered from the samples according to each media combination (enrichment broths and selective agars) is shown in Fig. 1.

All statistical analyses were performed in R (22) using RStudio Software (v.4.4.2). The “Pacman” package (23) was used for kappa coefficient calculations, and the “mcnemar.test” function was used for McNemar’s test. Friedman’s test and the pairwise post test were performed using “friedman.test” and “MCMRplus” package, respectively (24). Alpha diversity was determined using the Vegan package (v.2.6-4) (25). Confidence intervals (CIs) were calculated using 5,000 bootstrap replicates. The heatmap was generated using the “ComplexHeatmap” package (26).

## RESULTS

The overall *Salmonella* recovery considering the individual swabs was 53.5% (321 out of 600). Although there were no significant differences (*P* = 0.05784) across the prevalences observed in individual replicates: R1 = 53% (106 out of 200), R2 = 57.5% (115 out of 200), and R3 = 50% (100 out of 200), triplicate sampling yielded significantly higher *S. enterica* recovery rates (68.5%, 137 out of 200 samples) compared with individual replicates (*P* < 0.01; *P*_R1_ = 7.118e-8, *P*_R2_ = 7.562e-6, *P*_R3_ = 3.252e-9). Of all samples resulting in *Salmonella* recovery (*n* = 137), 77 (56.2%) were positive in all three replicates, while 30 (21.89%) were positive in two replicates and 30 (21.89%) in a single replicate. A moderate inter-replicate agreement (kappa = 0.598) was observed, indicating 30% data disagreement across the replicates.

Fourteen of the 137 positive samples were excluded from serovar diversity analysis due to processing, shipping, or sequencing issues, resulting in 123 analyzed samples (Table 1) from which isolates were submitted to whole-genome sequencing and *in silico* serovar identification. Considering the positive samples in multiple replicates (*n* = 93), only 26 (27.95%) showed identical serovar profiles across replicates.

**TABLE 1:** *Salmonella enterica* serovars identified in 123 surface water samples collected in triplicate by means of modified Moore swab (MMS)^*a*^

Sample ID	Replicate 1	Replicate 2	Replicate 3	Number of serovars	% serovar overlap
C122	N.D.	N.D.	Newport	1	0.0
C132	N.D.	N.D.	IV 50:z4,z23:-	1	0.0
C152	N.D.	N.D.	Infantis	1	0.0
C162	Rubislaw, Infantis	Saintpaul	Saintpaul	3	25.0
C171	Newport	Saintpaul	Javiana	3	0.0
C172	Schwarzengrund	Urbana	Rubislaw	3	0.0
C181	Muenster	Muenster	Michigan	2	33.3
C182	Corvallis, I4:b:-	München; Molade or Wippra; Kiambu	Rubislaw; Ohio; I4:b:-; Infantis	8	11.1
C191	I18:d:-	I18:d:-	I18:d:-	1	66.7
C192	I7:k:-	Javiana; Anatum	Carrau, Saintpaul	5	0.0
C193	Carrau	Saintpaul	Rubislaw	3	0.0
C222	Corvallis	Corvallis; Agona Sandiego	Carrau, Rubislaw, Hadar, Infantis	7	12.5
C223	Mbandaka	Infantis	Sandiego	3	0.0
C231	Gaminara	Muenchen	Muenchen, Javiana	3	25.0
C232	I16:e,h:e,n,z15	I16:e,h:e,n,z15	Schwarzengrund I16:e,h:e,n,z15	2	50.0
C252	N.D.	Gaminara	N.D.	1	0.0
C261	Corvallis	Anatum	Panama	3	0.0
C262	Schwarzengrund	Schwarzengrund	Schwarzengrund	1	66.7
C264	Muenchen, Infantis, Javiana	Rubislaw; Javiana; Muenchen; Infantis	Rubislaw, Javiana, Infantis	4	60.0
C272	Javiana	N.D.	N.D.	1	0.0
C281	N.D.	Saintpaul; Newport	Saintpaul, Newport	2	50.0
C292	N.D.	I18:d:-	N.D.	1	0.0
C362	Rubislaw	N.D.	N.D.	1	0.0
C364	Infantis	Infantis; Urbana	Rubislaw	3	25.0
C372	Muenchen	Muenchen	N.D.	1	50.0
C381	Muenchen	N.D.	N.D.	1	0.0
C391	I4:-:1,5	I4:-:1,5	N.D.	1	50.0
C3101	N.D.	Brandenburg	Brandenburg, Newport	2	33.3
C3102	N.D.	Brandenburg	N.D.	1	0.0
C3103	Othmarschen, Newport	Othmarschen	Othmarschen, Saintpaul	3	40.0
C411	Corvallis	Corvallis	Infantis	2	33.3
C413	Muenchen, Corvallis	Muenchen	IV6,7:z4,z24:-	3	25.0
C421	Infantis	Carrau	N.D.	2	0.0
C422	Corvallis	Infantis; I7:l,v:-	Corvallis	3	25.0
C431	Rubislaw; I7:l,v:-	Saintpaul; I4:b:-	Carrau; Sandiego; I7:l,v:-	6	14.3
C432	I16:r:e,n,z15	Carrau; Saintpaul	I16:r:e,n,z15	3	25.0
C441	Hadar	Hadar	Hadar	1	66.7
C442	Rubislaw	Rubislaw	Rubislaw	1	66.7
C443	N.D.	Poona	N.D.	1	0.0
C453	Saintpaul	Saintpaul	Saintpaul	1	66.7
C461	Newport	Newport	Newport	1	66.7
C462	N.D.	Businga	N.D.	1	0.0
C464	Corvallis, Muenchen	N.D.	N.D.	2	0.0
C481	Poona	Saintpaul	Newport	3	0.0
C482	Rubislaw	Saintpaul, Newport	Javiana, Newport	4	20.0
C4101	Carrau	Urbana	Newport	3	0.0
C4103	Javiana	Javiana, Infantis	Infantis	2	50.0
C512	Newport	Newport	Newport	1	66.7
C513	Newport	Newport; Panama	Hadar	3	25.0
C523	N.D.	I7:l,v:-	N.D.	1	0.0
C531	N.D.	Bullbay; Saintpaul	Bullbay; I -:l,v:e,n,z15	3	25.0
C532	Braenderup, Saintpaul	Saintpaul	N.D.	2	33.3
C551	N.D.	Infantis	N.D.	1	0.0
C552	Saintpaul	Saintpaul	Saintpaul	1	66.7
C561	Newport, Rubislaw	Newport; Rubislaw	Newport, Rubislaw	2	66.7
C562	N.D.	Poona	Poona	1	50.0
C582	Newport	Newport	Newport	1	66.7
C593	Newport	N.D.	Rubislaw	2	0.0
C621	Sandiego, Braenderup	N.D.	N.D.	2	0.0
C622	Braenderup, Hadar	Braenderup, Hadar, II42:r:-	Braenderup, Hadar	3	57.1
C623	I7:l,v:-	N.D.	Braenderup	2	0.0
C652	Gaminara	Gaminara	N.D.	1	50.0
C653	N.D.	IV[1],53:g,z51:-	N.D.	1	0.0
C664	N.D.	Urbana	Urbana, Braenderup	2	33.3
C671	N.D.	Poona	N.D.	1	0.0
C6103	Infantis	Infantis	Infantis	1	66.7
C722	Rubislaw, Meleagridis, Corvallis	Rubislaw; Saintpaul	N.D.	4	20.0
C751	N.D.	Carrau	N.D.	1	0.0
C762	N.D.	Saintpaul	N.D.	1	0.0
C7102	N.D.	Saintpaul	N.D.	1	0.0
C7103	N.D.	Saintpaul; Rubislaw	Saintpaul, Rubislaw	2	50.0
PC23	Rubislaw; IV43:z4,z23:-	N.D.	N.D.	2	0.0
1C21	Newport	Carrau	N.D.	2	0.0
1C51	Braenderup	N.D.	N.D.	1	0.0
1C53	N.D.	Carrau	N.D.	1	0.0
1C64	Rubislaw, Adelaide	Newport; Rubislaw	Newport	3	40.0
1C71	Rubislaw, Carrau	N.D.	N.D.	2	0.0
1C101	Oran	Oran; Poona	Oran	2	50.0
1C102	Oran	Oran	Oran	1	66.7
2C22	Javiana, Corvallis, Cerro	Corvallis, Cerro	Javiana, Cerro	3	50.0
2C23	Rubislaw	Carrau	Lomita; Rubislaw; I7:-:1,5	4	20.0
2C51	Braenderup	Rubislaw	N.D.	2	50.0
2C52	N.D.	N.D.	Muenster	1	0.0
2C53	Saintpaul	IV45:g,z51:- ; IV6,7:z4,z24:-	N.D.	3	0.0
2C61	Saintpaul	Rubislaw; Muenchen	Poona, Saintpaul	4	20.0
2C62	Poona	Muenchen	Schwarzengrund, Poona	4	25.0
2C64	Infantis	Infantis; IV43:z4,z23:-	Infantis; IV43:z4,z23:-	2	60.0
2C72	N.D.	Saintpaul, Corvallis	Saintpaul	2	33.3
2C102	Oran	Oran	Oran	1	66.7
2C103	Oran	Oran	Oran	1	66.7
3C21	Newport	Rubislaw, Saintpaul, Albany or Duesseldorf	Rubislaw	4	20.0
3C22	Corvallis, Agona, Albany or Duesseldorf	Albany or Duesseldorf	Agona	3	20.0
3C23	Agona; I3,10:d:-	Albany or Duesseldorf	Minnesota	4	0.0
3C51	Anatum	Carrau	Carrau	2	33.3
3C52	Saintpaul; Gaminara; I7:-:1,5	Saintpaul; I7:-:1,5	I7:-:1,5	3	42.9
3C62	N.D.	N.D.	Oran	1	0.0
3C64	Rubislaw, Poona	Rubislaw	Poona	2	50.0
3C102	Oran	Oran	Oran	1	66.7
3C103	Oran, Javiana	Saintpaul	Infantis	4	0.0
4C21	I4:d:-	N.D.	Rubislaw	2	0.0
4C22	Rubislaw, I4:d:-	Agona, Saintpaul	Javiana; II42:r:-	6	0.0
4C23	Hadar, Agona	Javiana, Poona	Anatum, Agona	5	16.7
4C51	Braenderup, Sandiego	Carrau, Agona, Braenderup	Carrau	4	33.3
4C52	Saintpaul; I7:-:1,5	Saintpaul, Muenchen	Saintpaul; Javiana; I7:-:1,5	4	42.9
4C53	Infantis; Saintpaul; IV43:z4,z23:-	Infantis	Infantis	3	40.0
4C61	Saintpaul	N.D.	N.D.	1	0.0
4C62	Saintpaul; I1,3,19:c:-	Poona, Inganda	Saintpaul, Oran	5	16.7
4C71	Michigan	Saintpaul	N.D.	2	0.0
4C72	Saintpaul	Saintpaul	N.D.	1	50.0
4C73	Rubislaw	Rubislaw	Rubislaw	1	66.7
4C101	Infantis, Rubislaw	Rubislaw	Oran, Rubislaw	3	40.0
4C102	Newport	Oran, Carrau	Oran, Pomona	4	40.0
4C103	Oran, Poona	Saintpaul, Oran	Infantis, Oran	4	50.0
5C22	N.D.	Agona	N.D.	1	0.0
5C23	Agona	N.D.	Sandiego	2	0.0
5C51	I7:-:1,5	N.D.	I7:-:1,5	1	50.0
5C52	Carrau; I7:-:1,5	Carrau; Sandiego; Agona; I7:-:1,5	Sandiego, Carrau, Thompson	5	50.0
5C53	N.D.	Farmsen or Poona, Saintpaul	Saintpaul	2	33.3
5C64	N.D.	Newport, Saintpaul	N.D.	2	0.0
5C72	Rubislaw	N.D.	N.D.	1	0.0
5C101	Oran	Oran	Oran	1	66.7
5C102	N.D.	Oran	Rubislaw, Oran	2	33.3
5C103	Infantis, Oran	Oran	Oran, Infantis	2	60.0

^*a*^ N.D., not detected.

**Fig 2:**
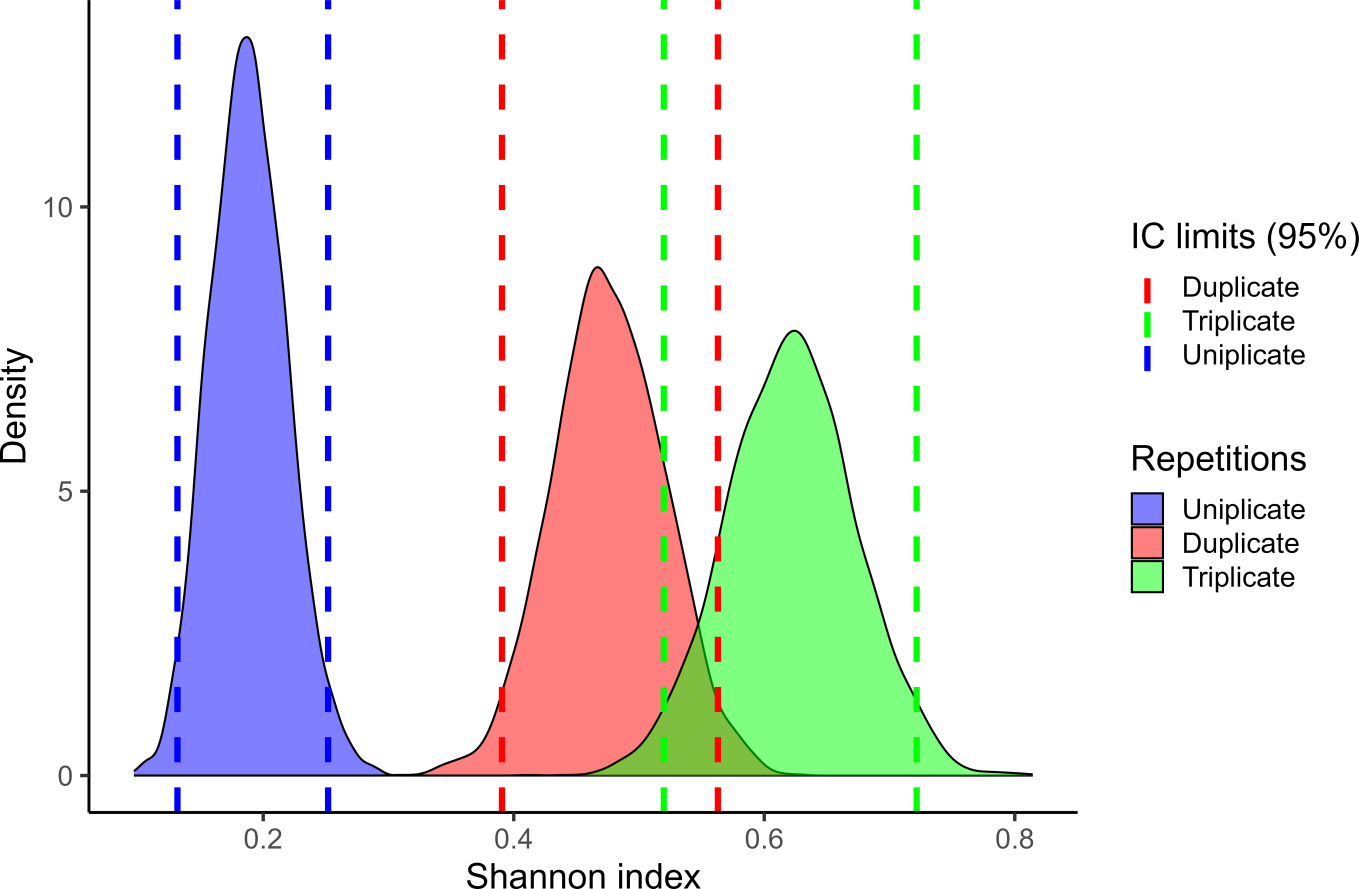
Shannon’s diversity index (*x* axis) of *Salmonella enterica* serovars recovered from surface waters by means of modified Moore swab in uniplicate, duplicate, and triplicate samplings.

According to alpha diversity analysis (Fig. 2), the mean richness index was significantly higher for triplicate (*S* = 3.2 ± 0.15) compared with duplicate (*S* = 2.8 ± 0.20) or single (*S* = 2.3 ± 0.30) samples, indicating that replicate sampling significantly increased the diversity of *S. enterica* detected in the samples. Pairwise post hoc analyses revealed significant differences between single and duplicate samplings (*P* < 0.01), and between single and triplicate samplings (*P* < 0.01). However, no significant differences (*P* > 0.01) were observed between duplicate and triplicate samplings. On average, single sampling missed approximately 1.15 serovars per sample compared to triplicate sampling.

Of the 200 samples, 174 were positive by BC-PCR, and from these, 39 (19.5%) were negative for *S. enterica* cultivation. Conversely, we recovered *S. enterica* from two BC-PCR-negative samples. The BC-PCR test failed to detect only 2 out of 137 culture-positive samples, resulting in 98.5% sensitivity compared with cultivation using triplicate sampling.

**Fig 3:**
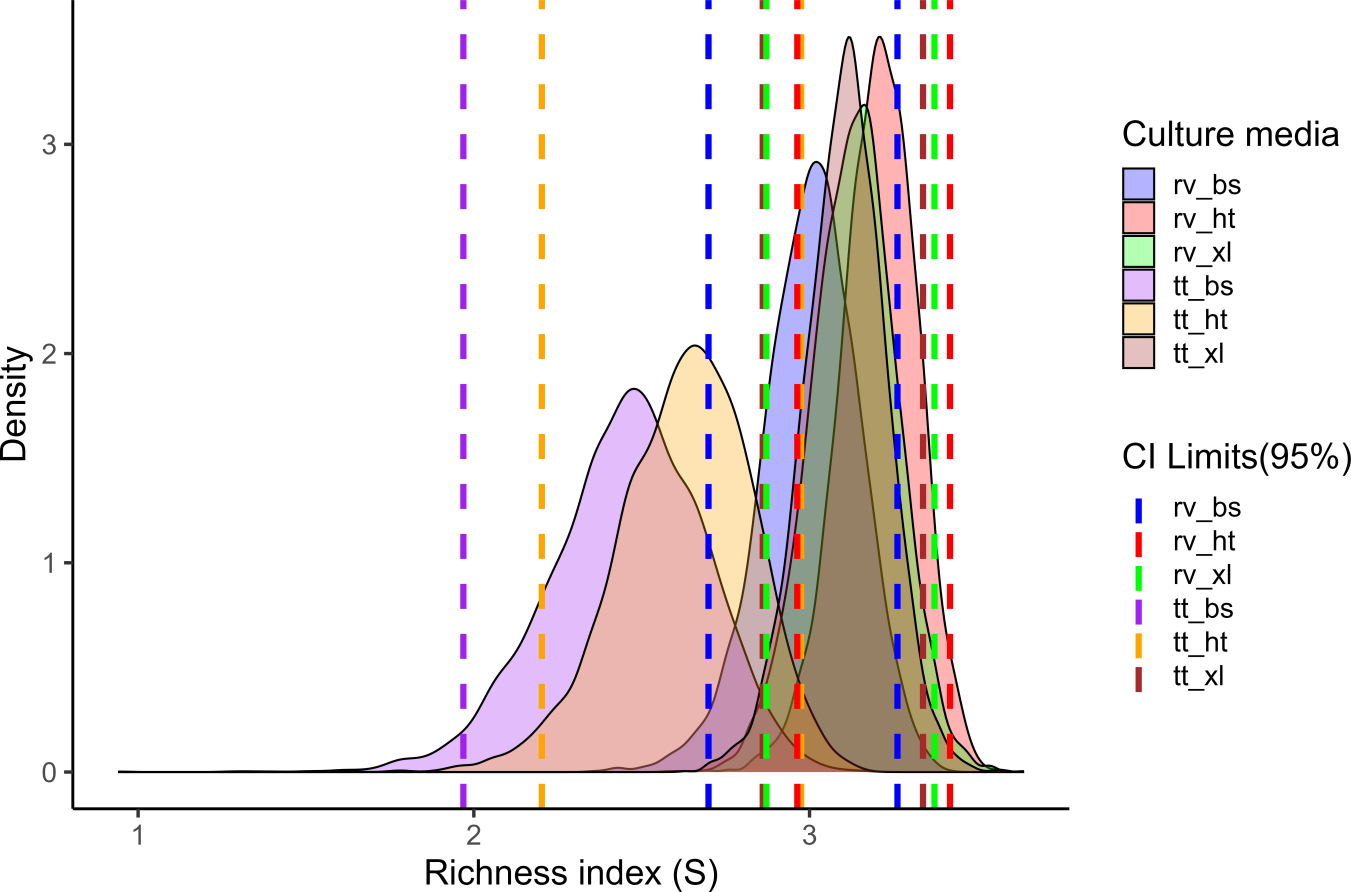
Shannon’s diversity index (*x* axis) of *Salmonella enterica* serovars recovered from surface water samples according to different enrichment broth (Rappaport-Vassiliadis [rv] and tetrathionate [tt]) and selective agars (xylose lactose tergitol [xl], Hektoen [ht], and bismuth sulfite [bs] combinations: rv_xl, tt_xl, rv_ht, tt_ht, rv_bs, and tt_bs.

Selective enrichment and plating media combinations significantly influenced serovar recovery patterns (Fig. 3). The use of RV enrichment broth resulted in higher diversity of *S. enterica* serovars according to the Shannon diversity index. In terms of selective agars, highest diversity was observed for XLT-4 agar: RV_XL (3.12, 95% CI: 2.874–3.370) and TT_XL (3.10, 95% CI: 2.857–3.341). RV_HT (3.19) slightly outperformed RV_XL (3.12) in diversity capture uniformity. Conversely, BS agar resulted in lower diversity, even though RV_BS (2.98) resulted in increased diversity compared with the TT_BS combination (2.42). Moderate agreement between RV and TT (89%, kappa = 0.636) suggested medium-specific serovar selectivity.

As shown in Fig. 1, RV and XLT-4 media demonstrated superior efficacy for serovar isolation. However, several serovars with single isolations (Thompson; Pomona; Molade/Wippra; Minnesota; Mbandaka; Lomita; Kiambu; Inganda; I 1,3,19:c:-; I-:I,v:e,n,z15; and Adelaide) were recovered only in specific enrichment broth-selective agar combinations.

## DISCUSSION

Although aquatic environments are not the primary habitat for *S. enterica*, our findings support recent studies indicating its high prevalence in natural surface water (16, 27), which represents a potential source of contamination in produce through irrigation systems. While individual sampling replicates resulted in similar *Salmonella* prevalences (53.0%, 57.5%, and 50.0%), the combined results from triplicate sampling yielded 68.5% prevalence, representing 11% and 18.5% increases in *S. enterica* recovery compared with duplicate and uniplicate samplings, respectively.

Both duplicate and triplicate samplings improved the diversity of *S. enterica* serovars recovered from water samples compared with the uniplicate sampling, which missed approximately 1.15 serovars per sample. Notably, this increase in serovar diversity associated with replicate sampling was significant despite the majority of our samples originating from stagnant water. Theoretically, replicate sampling might be even more valuable when sampling flowing water, such as rivers.

Importantly, we did not employ any genotypic or phenotypic tests to screen isolates before sequencing and serovar identification. Although the use of an O antisera panel or repetitive element sequence-based PCR could serve as an interesting alternative to enhance serovar detection, the associated increases in time, costs, and labor requirements could compromise their practical utility for routine monitoring purposes.

While our current methodology precluded quantitative correlation between positive swabs and *Salmonella* abundance or serovar diversity in environmental samples, this represents an important avenue for future research. We believe that replicate sampling could be crucial for samples with low numbers of *S. enterica*. This assumption is further supported by our previous seasonal study in the region, indicating rainfall as the strongest predictor of *S. enterica* recovery compared with samples collected in dry periods, potentially harboring lower bacterial loads that would benefit from enhanced sampling strategies (28).

In this regard, we suggest that further studies on the prevalence and serovar diversity of *S. enterica* in water should incorporate quantitative analysis in parallel, through enumeration using conventional microbiology techniques or alternative absolute quantification approaches, such as droplet digital PCR systems.

The inability to isolate *S. enterica* from 39 PCR-positive broths (19.5%) is often attributed to BC-PCR detecting non-viable organisms. However, these results likely stem from a substantial disparity between initial sample volumes used in cultivation (∼10 µL) versus BC-PCR (∼1 mL), a factor frequently overlooked in comparative studies. Although two culture-positive samples had negative BC-PCR results, possibly due to inhibitors, the high sensitivity rate (98.54%) for viable *Salmonella* detection supports BC-PCR as a useful, cost-effective screening method for *Salmonella* contamination in environmental water samples.

On the other hand, isolation failure in BC-PCR-positive samples could also be due to the inability of certain *S. enterica* serovars to grow on some media. Although the use of multiple culture media in our study proved essential for comprehensive *Salmonella* serovar recovery from environmental water samples, it is possible that the addition of other media combinations could lead to the recovery of more serovars.

Our findings support previous studies showing that serovar recovery patterns can be influenced by different selective media and specific incubation conditions (29, 30). While TT-XLT was an effective combination for recovering *S. enterica*, TT-HT and TT-BS combinations resulted in significantly lower detection rates. Successful recovery on these media occurred primarily for samples that were also positive under the TT-XLT combination, suggesting limited benefit from adding HT and BS to TT-enriched samples.

Notably, we obtained single isolations for some serovars that grew exclusively with a specific enrichment broth-agar combination, even though the sample was cultivated using five other media combinations. For example, *S*. Inganda and *S*. I 1,3,19:c:- were isolated solely from TT-BS and TT-HT, while *S*. Lomita and *S*. Pomona were recovered exclusively from RV-HT.

Significant differences were observed between RV and TT broths when associated with the same agars. Overall, RV-enriched samples resulted in superior performance compared with those enriched in TT, corroborating a previous study investigating various types of samples, including water (31). For instance, *S*. Javiana and *S*. Corvalis tended to be preferentially isolated from RV-enriched samples. We believe that the low number of *Salmonella* cells in natural water environments explains the high recovery rates in favor of RV. The better performance is mainly attributed to superior competitor suppression compared to TT, as reported in both classic controlled kinetic experiments (32) and field surveys (31). This feature is crucial for samples with low *S. enterica* numbers and the presence of abundant competitors, such as environmental samples.

Importantly, unlike clinical samples, multiple strains and serovars are commonly found in natural surface waters, as observed in our study. According to a genomic investigation in Brazil, Chile, and Mexico, the number of non-clonal isolates in surface water samples ranged from 1 to 10 (19). Moreover, this same study revealed that clonally related isolates were detected in samples collected up to 3 years apart, suggesting the long-term persistence of specific strains. Therefore, the use of an appropriate sampling method that accurately captures the intrinsic *S. enterica* diversity in water is crucial for the success of epidemiological investigations and monitoring initiatives. In this aspect, media selection must also consider the dominance of certain serovars over others, depending on the media employed, probably due to varying susceptibility among *Salmonella* serovars to the inhibitory compounds in selective media. This has been well documented in a controlled trial (30) in which dominant *S. enterica* serovars were recovered from mixed cultures. Interestingly, in this same study, the use of a more nutrient-rich version of RV, such as Rappaport-Vassiliadis soya peptone broth, led to different patterns of *S. enterica* strains emerging from mixed cultures compared with a conventional RV formula (30). These results not only highlight the importance of using multiple enrichment broths, as previously suggested (33, 34), but also encourage further research toward the selection of the most appropriate media for accurate surveillance of *S. enterica* serovars in natural water bodies. Such studies should preferably include quantitative approaches for *Salmonella* enumeration, such as droplet-digital PCR, and microbial background information through 16S rRNA metabarcoding or shotgun metagenomics.

The capacity of the MMS sampling method to process larger water volumes (10 L) offers distinct advantages over conventional approaches (14). While ultrafiltration could potentially enhance detection sensitivity, MMS represents a cost-effective, logistically viable, and reliable field sampling method (11). When considering MMS application to sample volumes exceeding 10 L, researchers should weigh using a single filtration cassette unit versus true replicates, accounting for technical limitations such as potential swab clogging. Multiple 10 L replicates may provide superior accuracy compared to single larger-volume samples, though practical constraints must be considered. While triplicate sampling significantly increases laboratory workload, logistical complexity, and processing costs, our results demonstrate its value in enhancing both detection frequency and serovar diversity. As a practical alternative, duplicate sampling emerges as a viable compromise compared with triplicate sampling, offering comparable statistical power with reduced resource requirements.

In conclusion, replicate MMS sampling significantly improves the accuracy of prevalence assessment and diversity characterization of *S. enterica* serovars in surface waters compared with single samples. However, the implementation of sampling replicates should be evaluated based on specific project requirements, considering factors such as budget constraints and workload capacity, to optimize *S. enterica* surveillance protocols in aquatic environments.

## SUPPLEMENTAL MATERIAL

Table S1 aem.00647-25-s0001.xlsx Full identification of surface water samples and sampling sites: watershed basins, location, water source, season, sampling date and time.

Table S2 aem.00647-25-s0002.xlsx Genome accession identification in NCBI and respective *Salmonella enterica* serovars recovered in each sampling replicate.

ASM does not own the copyrights to Supplemental Material that may be linked to, or accessed through, an article. The authors have granted ASM a non-exclusive, world-wide license to publish the Supplemental Material files. Please contact the corresponding author directly for reuse.

## References

[1] Roth GA, Abate D, Abate KH, Abay SM, Abbafati C, Abbasi N, Abbastabar H, Abd-Allah F, Abdela J, Abdelalim A, et al.. 2018. Global, regional, and national age-sex-specific mortality for 282 causes of death in 195 countries and territories, 1980–2017: a systematic analysis for the Global Burden of Disease Study 2017. The Lancet 392:1736–1788. doi:10.1016/S0140-6736(18)32203-7 PMC6227606 30496103

[2] Andrews-Polymenis HL, Bäumler AJ, McCormick BA, Fang FC. 2010. Taming the elephant: Salmonella biology, pathogenesis, and prevention. Infect Immun 78:2356–2369. doi:10.1128/IAI.00096-10 20385760 PMC2876576

[3] Walsh KA, Bennett SD, Mahovic M, Gould LH. 2014. Outbreaks associated with cantaloupe, watermelon, and honeydew in the United States, 1973-2011. Foodborne Pathog Dis 11:945–952. doi:10.1089/fpd.2014.1812 25407556 PMC4627691

[4] Allard SM, Callahan MT, Bui A, Ferelli AMC, Chopyk J, Chattopadhyay S, Mongodin EF, Micallef SA, Sapkota AR. 2019. Creek to Table: tracking fecal indicator bacteria, bacterial pathogens, and total bacterial communities from irrigation water to kale and radish crops. Sci Total Environ 666:461–471. doi:10.1016/j.scitotenv.2019.02.179 30802661

[5] Interagency Food Safety Analytics Collaboration. 2022. Foodborne illness source attribution estimates for Salmonella, Escherichia coli O157, and Listeria monocytogenes – United States, 2022. GA and D.C.: U.S. Department of Health and Human Services, Centers for Disease Control and Prevention, Food and Drug Administration, U.S. Department of Agriculture’s Food Safety and Inspection Service. Available from: https://www.cdc.gov/ifsac/php/data-research/annual-report-2022.html. Retrieved 13 Dec 2024.

[6] Liu H, Whitehouse CA, Li B. 2018. Presence and persistence of Salmonella in water: the impact on microbial quality of water and food safety. Front Public Health 6:159. doi:10.3389/fpubh.2018.00159 29900166 PMC5989457

[7] U.S. Food & Drug Administration. 2024. Outbreak investigation of Salmonella: cucumbers (June 2024). Available from: https://ww.fda.Gov/Food/Outbreaks-Foodborne-Illness/Outbreak-Investigation-Salmonella-Cucumbers-June-2024. Retrieved 19 May 2025.

[8] Martínez MC, Retamal P, Rojas-Aedo JF, Fernández J, Fernández A, Lapierre L. 2017. Multidrug-resistant outbreak-associated Salmonella strains in irrigation water from the Metropolitan Region, Chile. Zoonoses Public Health 64:299–304. doi:10.1111/zph.12311 27860367

[9] Santo Domingo JW, Harmon S, Bennett J. 2000. Survival of Salmonella species in river water. Curr Microbiol 40:409–417. doi:10.1007/s002840010079 10827285

[10] Liao C-H, Shollenberger LM. 2003. Survivability and long-term preservation of bacteria in water and in phosphate-buffered saline. Lett Appl Microbiol 37:45–50. doi:10.1046/j.1472-765x.2003.01345.x 12803555

[11] Sharma M, Handy ET, East CL, Kim S, Jiang C, Callahan MT, Allard SM, Micallef S, Craighead S, Anderson-Coughlin B, et al.. 2020. Prevalence of Salmonella and Listeria monocytogenes in non-traditional irrigation waters in the Mid-Atlantic United States is affected by water type, season, and recovery method. PLoS One 15:e0229365. doi:10.1371/journal.pone.0229365 32182252 PMC7077874

[12] Acheamfour CL, Parveen S, Hashem F, Sharma M, Gerdes ME, May EB, Rogers K, Haymaker J, Duncan R, Foust D, et al.. 2021. Levels of Salmonella enterica and Listeria monocytogenes in alternative irrigation water vary based on water source on the Eastern Shore of Maryland. Microbiol Spectr 9:e0066921. doi:10.1128/Spectrum.00669-21 34612697 PMC8510256

[13] Kahler A, Johnson T, Hahn D, Narayanan J, Derado G, Hill V. 2015. Evaluation of an ultrafiltration-based procedure for simultaneous recovery of diverse microbes in source waters. Water (Basel) 7:1202–1216. doi:10.3390/w7031202 26530003 PMC4627901

[14] Sbodio A, Maeda S, Lopez-Velasco G, Suslow TV. 2013. Modified Moore swab optimization and validation in capturing E. coli O157:H7 and Salmonella enterica in large volume field samples of irrigation water. Food Res Int 51:654–662. doi:10.1016/j.foodres.2013.01.011

[15] Thrusfield M. 2018. Veterinary epidemiology. John Wiley & Sons.

[16] Rocha AD de L, Ferrari RG, Pereira WE, de Lima LA, Givisiez PEN, Moreno-Switt AI, Toro M, Delgado-Suárez EJ, Meng J, de Oliveira CJB. 2022. Revisiting the biological behavior of Salmonella enterica in hydric resources: a meta-analysis study addressing the critical role of environmental water on food safety and public health. Front Microbiol 13:802625. doi:10.3389/fmicb.2022.802625 35722289 PMC9201643

[17] Andrews WH, Wang H, Jacobson A, Ge B, Zhang G, Hammack T. 2024. “Salmonella” in bacteriological analytical manual (BAM). U.S. Food and Drug Administration

[18] Freschi CR, Carvalho LF de O e S, Oliveira CJB de. 2005. Comparison of DNA-extraction methods and selective enrichment broths on the detection of Salmonella Typhimurium in swine feces by polymerase chain reaction (PCR). Braz J Microbiol 36:363–367. doi:10.1590/S1517-83822005000400011

[19] Chen Z, Moreno-Switt AI, Reyes-Jara A, Delgado Suarez E, Adell AD, Oliveira CJB, Bonelli RR, Huang X, Brown E, Allard M, Grim C, Bell R, Meng J, Toro M. 2024. A multicenter genomic epidemiological investigation in Brazil, Chile, and Mexico reveals the diversity and persistence of Salmonella populations in surface waters. MBio 15:e0077724. doi:10.1128/mbio.00777-24 38920393 PMC11253603

[20] Landis JR, Koch GG. 1977. The measurement of observer agreement for categorical data. Biometrics 33:159. doi:10.2307/2529310 843571

[21] McHugh ML. 2012. Interrater reliability: the kappa statistic. Biochem Med (Zagreb) 22:276–282. 23092060 PMC3900052

[22] R Core Team. 2021. R: a language and environment for statistical computing. R Foundation for Statistical Computing, Vienna, Austria. https://www.R-project.org.

[23] Rinker T, Kurkiewicz D. 2015. Pacman: package management tool. CRAN: contributed packages. 10.32614/CRAN.package.pacman.

[24] Pohlert T. 2018. PMCMRplus: calculate pairwise multiple comparisons of mean rank sums extended. CRAN: contributed packages. 10.32614/CRAN.package.PMCMRplus.

[25] Oksanen J, Simpson GL, Blanchet FG, Kindt R, Legendre P, Minchin PR, et al.. 2001. Vegan: community ecology package. CRAN: contributed packages. 10.32614/CRAN.package.vegan.

[26] Gu Z, Eils R, Schlesner M. 2016. Complex heatmaps reveal patterns and correlations in multidimensional genomic data. Bioinformatics 32:2847–2849. doi:10.1093/bioinformatics/btw313 27207943

[27] Bell RL, Zheng J, Burrows E, Allard S, Wang CY, Keys CE, Melka DC, Strain E, Luo Y, Allard MW, Rideout S, Brown EW. 2015. Ecological prevalence, genetic diversity, and epidemiological aspects of Salmonella isolated from tomato agricultural regions of the Virginia Eastern Shore. Front Microbiol 6:415. doi:10.3389/fmicb.2015.00415 25999938 PMC4423467

[28] Rocha ADL, Lima LA, Sales GFC, Silva NJ, Gomes MLR, Pereira WE, Givisiez PEN, Brown EW, Allard MW, Bell RL, Toro M, Meng J, Oliveira CJB de. 2025. Predictors of Salmonella enterica contamination in agricultural and livestock-impacted natural watersheds. Environ Pollut 371:125782. doi:10.1016/j.envpol.2025.125782 39894151

[29] Obe T, Berrang ME, Cox NA, House SL, Shariat NW. 2021. Comparison of selective enrichment and plating media for Salmonella isolation from broiler carcasses. J Food Saf 41. doi:10.1111/jfs.12928

[30] Gorski L. 2012. Selective enrichment media bias the types of Salmonella enterica strains isolated from mixed strain cultures and complex enrichment broths. PLoS One 7:e34722. doi:10.1371/journal.pone.0034722 22496847 PMC3319605

[31] Gorski L, Parker CT, Liang A, Cooley MB, Jay-Russell MT, Gordus AG, Atwill ER, Mandrell RE. 2011. Prevalence, distribution, and diversity of Salmonella enterica in a major produce region of California. Appl Environ Microbiol 77:2734–2748. doi:10.1128/AEM.02321-10 21378057 PMC3126348

[32] Rhodes P, Quesnel LB. 1986. Comparison of Muller-Kauffmann tetrathionate broth with Rappaport-Vassiliadis (RV) medium for the isolation of salmonellas from sewage sludge. J Appl Bacteriol 60:161–167. doi:10.1111/j.1365-2672.1986.tb03374.x 3700279

[33] Pal A, Marshall DL. 2009. Comparison of culture media for enrichment and isolation of Salmonella spp. from frozen Channel catfish and Vietnamese basa fillets. Food Microbiol 26:317–319. doi:10.1016/j.fm.2008.12.003 19269575

[34] Hughes D, Dailianis AE, Hill L, Curiale MS, Gangar V, Arnold D. 2003. Salmonella in foods: new enrichment procedure for TECRA Salmonella visual immunoassay using a single RV(R10) only, TT only, or dual RV(R10) and TT selective enrichment broths (AOAC official method 998.09): collaborative study. J AOAC Int 86:775–790. 14509439

